# A Molecular Dynamics Study on Cutting-Strategy-Dependent Subsurface Damage in Single-Crystal Silicon During Ultra-Precision Machining

**DOI:** 10.3390/mi17070872

**Published:** 2026-07-22

**Authors:** Bo Huang, Pengyue Zhao, Liang Qiao, Ruihan Li, Meng Li, Shuhan Peng, Huan Liu

**Affiliations:** 1 Heilongjiang Provincial Meteorological Data Center, No. 71, Tietan Street, Harbin 150001, China; bohuang_129@163.com (B.H.); lqiao_122@163.com (L.Q.); pengsh_11@163.com (S.P.); 2 School of Instrumentation Science and Engineering, Harbin Institute of Technology, Harbin 150080, China; pyzhao@hit.edu.cn (P.Z.); ruihanli@hrbeu.edu.cn (R.L.); 25b901080@stu.hit.edu.cn (M.L.)

**Keywords:** monocrystalline silicon, molecular dynamics, ultra-precision machining, multi-pass cutting, subsurface damage

## Abstract

This study investigates the material removal mechanism and the evolution of subsurface damage (SSD) in single-crystal silicon during ultra-precision machining using molecular dynamics (MD) simulations. A three-dimensional MD model was established by employing Tersoff and Morse interaction potentials to evaluate the effects of different cutting strategies on cutting response, stress distribution, surface morphology, and defect evolution. The results show that the multi-pass cutting strategy effectively reduces the mean cutting force and suppresses severe stress concentration regions exceeding 7 GPa. This improvement is mainly attributed to the progressive release of residual stress and the more gradual removal of material during successive cutting passes. The formation of SSD is dominated by lattice distortion and amorphous phase transformation, both of which are closely associated with localized high von Mises stress beneath the machined surface. Further analyses of surface morphology and defect density indicate that a multi-pass strategy with a single-pass cutting depth below 1 nm provides a favorable balance between machining efficiency and surface integrity. These findings provide atomistic insights into damage suppression and process optimization for the ultra-precision machining of brittle semiconductor materials.

## 1. Introduction

Single-crystal silicon is a fundamental material in semiconductor manufacturing, microelectromechanical systems, and precision optical components because of its excellent electronic, optical, and thermal properties [1,2]. However, its strong covalent bonding, high hardness, and intrinsic brittleness make it highly susceptible to defect formation during nanometric ultra-precision machining [3,4]. At the atomic scale, material removal in silicon is governed by the coupled evolution of local stress, lattice distortion, phase transformation, and surface fracture. These processes may lead to irregular chip accumulation, exit burr formation, and subsurface damage (SSD), thereby degrading dimensional accuracy, surface integrity, and device reliability [5]. Experiments often fail to capture transient deformation and damage events. Consequently, molecular dynamics (MD) simulation has become an effective approach for revealing atomistic mechanisms, including lattice disordering, amorphization, stress localization, and the brittle-to-ductile transition during nanoscale cutting [6,7,8]. Therefore, a systematic understanding of how different cutting strategies regulate cutting force fluctuation, local stress concentration, and SSD evolution is essential for improving the ultra-precision machining quality of brittle semiconductor ma terials [9].

MD simulations have been widely applied to investigate the material removal behavior, surface integrity, and SSD evolution of single-crystal silicon under nanoscale machining conditions [10,11]. In terms of multi-pass cutting, Wu et al. [12] examined the evolution of SSD during successive diamond tool passes, with emphasis on the spatial development of amorphous layers and shear strain. Dai et al. [13,14] further compared atom migration in shear zones between overlapping multi-pass cutting and single deep cutting and analyzed the rheological response and strain hardening behavior induced by multigrain multi-pass interference. From the perspective of process parameters, Zhao et al. [7,15,16] investigated the effects of cutting modes, cutting depth, and machining sequence on removal rate, hydrostatic pressure, elastic recovery, and structural integrity, and established process window distributions for predicting edge morphology evolution based on local stress states. Li et al. [17] focused on edge morphology control and clarified the influence of cutting strategies on high-stress atom accumulation, exit burr formation, and SSD development. For transient boundary phenomena, Geng et al. [18] demonstrated that MD simulation can capture unstable burr fracture processes that are difficult to observe experimentally. In addition, stress-related mechanisms have been explored from different perspectives. Olufayo et al. [19] indicated that multi-pass cutting can reduce stress concentration and suppress the accumulation of high-stress atoms, whereas Ai et al. [20] linked static stress fields with dynamic topological evolution to explain the formation of exit burrs. Zhang et al. [21] revealed stress distribution and phase transformation pathways from pristine silicon to high-pressure and amorphous phases, while Guo et al. [22] introduced statistical descriptions of high-stress atom populations. Tanaka and Shimada [23] interpreted burr suppression in multi-pass cutting based on shear slip and brittle-to-ductile transition, and Guo et al. [24] further explained the regulatory effect of multi-pass machining from the viewpoint of stress relaxation. More recently, the coupling effects of depth sequence, anisotropy, energy dissipation, friction, surface texture, and external field assistance have also been considered [25,26,27,28,29].

Although considerable progress has been made in understanding nanoscale cutting of silicon, the atomistic mechanisms by which dynamic multi-pass strategies with different depth sequences regulate local stress accumulation, exit burr instability, and SSD evolution remain insufficiently clarified. Existing studies have mainly focused on single-pass cutting, fixed cutting depths, or isolated process parameters. In contrast, the spatial redistribution of high-stress atoms, residual stress release, and damage suppression behavior under continuous-depth gradient strategies have not been fully established. In particular, it remains unclear whether reducing the single-pass depth can monotonically improve surface integrity, or whether an optimal depth configuration exists in which stress relaxation and material removal efficiency are balanced.

In this study, three-dimensional MD simulations were performed to investigate the ultra-precision cutting response of single-crystal silicon under different cutting strategies. The purpose of this work lies in the controlled comparison of four cutting strategies with different pass numbers and depth partitioning while maintaining the same total removal depth of 2 nm. This design isolates the effects of pass arrangement and single-pass cutting depth from that of the total removal depth and enables their coupled influences on cutting force, von Mises stress distribution, high-stress atom accumulation, exit burr formation, and SSD evolution to be systematically evaluated. The results reveal a nonmonotonic relationship between depth configuration and high-stress atom accumulation at shallow cutting depths. Moreover, an optimized multi-pass strategy is shown to transform the burr formation mechanism from stress concentrated fracture to more stable plastic flow. This work provides atomistic evidence for understanding damage suppression in silicon nanomachining and offers theoretical guidance for the ultra-precision manufacturing of brittle semiconductor materials.

## 2. Simulation Methods

### 2.1. Simulation Model

**Figure 1:**
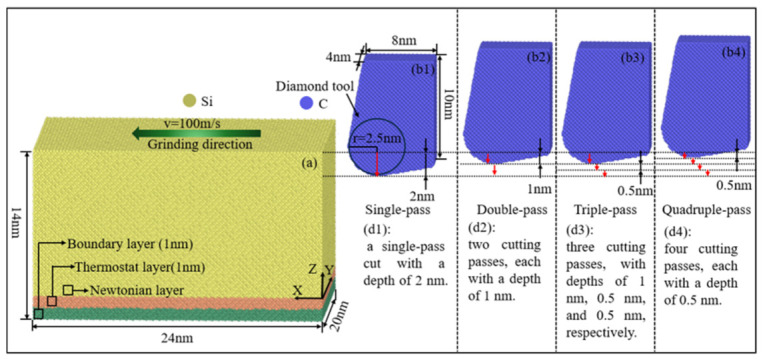
(**a**) Atomic-scale schematic of the MD simulation model for the ultra-precision cutting process of monocrystalline silicon, detailing the tool–workpiece interaction and the specific cutting depth configurations for (**b1**) single-pass, (**b2**) double-pass, (**b3**) triple-pass, and (**b4**) quadruple-pass strategies. The notations d1–d4 denote the single-pass, double-pass, triple-pass, and quadruple-pass strategies, respectively. The red arrows indicate the downward infeed for successive passes, and the black double-headed arrows indicate the depth of cut per pass. The solid horizontal line represents the initial workpiece surface, the horizontal dashed lines represent the target surface levels after successive passes, and the vertical dashed lines separate the cutting strategies.

To elucidate the effects of cutting mode and depth sequence on the material removal behavior of single-crystal silicon, MD simulations were performed to reproduce the nanoscale cutting process at the atomic level. Figure 1 shows the MD model for nanomachining of single-crystal silicon, which consists of a diamond cutting tool and a silicon workpiece. The silicon workpiece was constructed in the diamond-cubic structure with a lattice constant of 0.543 nm. The crystallographic orientation of the single-crystal silicon workpiece was defined such that the X, Y, and Z axes were parallel to the [100], [010], and [001] crystallographic directions, respectively. Accordingly, the upper machined surface corresponds to the Si(001) plane, and the cutting direction along the X axis is parallel to the [100] crystallographic direction. The workpiece dimensions were 24 nm × 20 nm × 14 nm, corresponding to a total of 384,356 atoms. The diamond tool had dimensions of 8 nm × 4 nm × 10 nm, and the cutting edge radius was 2.5 nm. Before machining, the system was first relaxed to obtain a stable initial configuration. Periodic boundary conditions were applied in the lateral, longitudinal, and vertical directions during the initial relaxation stage. The total energy of the system was minimized using the conjugate gradient method, followed by equilibration for 50 ps under an isothermal isobaric (NPT) ensemble to achieve a thermodynamically stable state. A time step of 1 fs was used for all dynamic simulation stages.

During the cutting stage, the boundary conditions were changed to nonperiodic in the lateral and height directions, while periodicity was retained in the longitudinal direction perpendicular to the cutting plane. Due to the inherent scale limitations of MD simulations, coupled with considerations of time and computational cost, a cutting speed of 100 m/s was employed in this study to investigate the effects of the number of cutting passes and the depth-of-cut sequence. This velocity has been validated and utilized in prior MD studies [15,18]. The silicon workpiece was divided vertically into three functional regions, namely the Newtonian layer, the thermostat layer, and the boundary layer. During cutting, atoms in the Newtonian layer were integrated using the velocity Verlet algorithm under the microcanonical (NVE) ensemble [30]. Atoms in the thermostat layer were integrated using the same algorithm and coupled to a Berendsen thermostat at 293 K, corresponding to NVT-type temperature control [31]. The bottom boundary atoms were fixed throughout the simulation to provide mechanical support and mimic the constraint imposed by the bulk substrate.

Four cutting strategies were designed to examine the influence of multi-pass cutting and depth sequence on the machining response. The total removal depth was fixed at 2 nm for all cases to maintain the same target removal depth and to avoid the confounding effect of differences in the total cutting depth. Meanwhile, a total depth of 2 nm can be precisely partitioned into incremental cuts of 1 nm and 0.5 nm. In the single-pass strategy, denoted as d1, the cutting depth was set to 2 nm. In the double-pass strategy, denoted as d2, two successive cuts were performed with a depth of 1 nm for each pass. In the triple-pass strategy, denoted as d3, the cutting depths were 1 nm, 0.5 nm, and 0.5 nm, respectively. In the quadruple-pass strategy, denoted as d4, four successive cuts were performed with a cutting depth of 0.5 nm for each pass. Therefore, the total nominal removal depth was maintained at 2 nm for all cutting strategies, allowing the effect of pass number and single-pass depth to be isolated.

After cutting, the system was further relaxed to stabilize the machined workpiece and reduce nonequilibrium thermal fluctuations. The relaxed configurations were then used to quantify the local stress distribution, the number and spatial distribution of high-stress atoms, phase transformation characteristics, subsurface damage evolution, and exit burr morphology. These analyses provide a basis for revealing the relationship between multi-pass cutting strategy, stress redistribution, and damage formation in single-crystal silicon nanomachining.

### 2.2. Potential Function

The reliability of MD simulations strongly depends on the ability of the selected interatomic potentials to describe atomic interactions under large deformation and high-contact-stress conditions. In the present silicon nanomachining model, the atomic interactions mainly include three parts: Si–Si interactions within the single-crystal silicon workpiece, C–C interactions within the diamond tool, and C–Si interactions at the tool–workpiece interface. To balance physical accuracy and computational efficiency, a hybrid Tersoff–Morse potential framework was adopted in this study. This is because the Tersoff potential accurately captures the complex structural transformations and covalent bond breaking within the silicon lattice, while the Morse potential effectively models the heterogeneous nonlinear contact and friction dynamics at the tool–workpiece interface.

The covalent interactions among silicon atoms in the workpiece were described using the Tersoff potential [32]. The Tersoff potential is a many-body bond order potential that accounts for the dependence of bond strength on the local atomic environment and coordination state. Therefore, it is suitable for describing directional covalent bonding, bond breaking, bond reforming, lattice distortion, and defect formation in single-crystal silicon during nanoscale cutting. In this study, the Tersoff potential provides the physical basis for analyzing stress-induced lattice disordering, amorphization, and SSD evolution in the silicon workpiece.

The carbon atoms in the diamond tool were also described using the Tersoff potential to define the covalent bonding framework of the diamond structure. However, because diamond has a much higher hardness and elastic modulus than single-crystal silicon, the tool was treated as a rigid body during the cutting process. Under this assumption, the relative positions of carbon atoms in the tool remain unchanged, and the deformation response is mainly concentrated in the silicon workpiece. This treatment reduces computational cost and is commonly used in MD simulations of nanocutting when the main focus is the deformation, removal, and damage evolution of the workpiece.

The interaction between the diamond tool and the silicon workpiece was described using the Morse potential, following a previous molecular dynamics study of nanometric machining of single-crystal silicon using a rigid diamond tool [19]. The Morse potential is an empirical pair potential that can represent the nonlinear attractive and repulsive interactions at the heterogeneous C–Si contact interface. The potential energy between interfacial atoms is expressed as follows:

(1) $$E(r)=De\left[e^{-2\alpha (r-re)}-2e^{-\alpha (r-re)}\right]$$

where *E*(*r*) is the interatomic potential energy, *r* is the distance between two interacting atoms, *De* is the bond dissociation energy, α controls the width of the potential well, and *re* is the equilibrium bond length. The Morse potential parameters were set as De = 0.435 eV, α = 4.6487 Å^−1^, and re = 1.9475 Å. This parameterization is suitable for the present model because the diamond tool is treated as a rigid body and the Morse potential is used primarily to describe the short-range attractive and repulsive interactions at the mechanical C–Si contact interface. These parameters define the interfacial contact, adhesion, friction, and ploughing interactions between the diamond cutting edge and the silicon lattice.

The hybrid Tersoff–Morse potential framework was implemented in LAMMPS [33]. In this framework, the Tersoff potential governs the covalent bonding and structural evolution within the silicon workpiece and diamond tool, whereas the Morse potential describes the heterogeneous contact interaction between carbon and silicon atoms. This potential setting enables the simulation to capture the atomic-scale material removal process, local stress redistribution, lattice disordering, amorphous transformation, high-stress atom accumulation, and exit burr formation during multi-pass ultra-precision cutting of single-crystal silicon.

## 3. Results and Discussion

### 3.1. Surface Morphology and Material Removal Analysis

**Figure 2:**
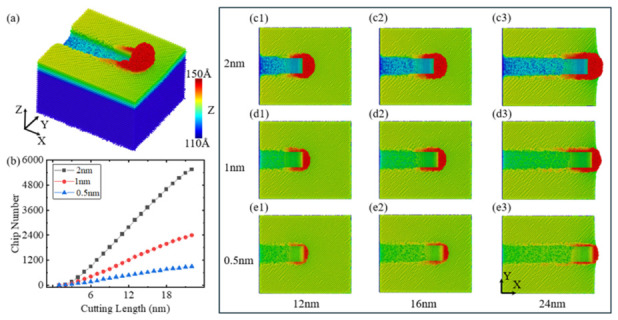
Surface chips of monocrystalline silicon under different cutting depths in ultra-precision machining: (**a**) substrate model; (**b**) statistical results of surface chip quantity; (**c1**–**c3**) cutting depth of 2 nm; (**d1**–**d3**) cutting depth of 1 nm; (**e1**–**e3**) cutting depth of 0.5 nm.

Figure 2 shows the material removal behavior of single-crystal silicon under different cutting depths during ultra-precision machining. As shown in Figure 2(c1–e3), chips are mainly generated in front of and on both sides of the diamond tool at cutting depths of 2 nm, 1 nm, and 0.5 nm. This phenomenon indicates that the region ahead of the cutting edge acts as the primary stress concentration zone, where the silicon lattice first undergoes local yielding, disordering, and material separation. Meanwhile, the formation of lateral chips on both sides of the machined groove suggests that a considerable portion of the displaced atoms are extruded sideways under the combined action of compression and shear. As the cutting distance increases, the number of removed atoms gradually increases, confirming that material removal in nanoscale cutting is a progressive process involving continuous lattice distortion, atomic extrusion, and chip accumulation. The quantitative results in Figure 2b further reveal two clear trends. First, the number of removed atoms increases continuously with cutting distance, reflecting the accumulation of material removal during tool advancement. Second, the removed volume increases with cutting depth. A larger cutting depth leads to deeper tool penetration, affects more atomic layers, enlarges the plastic deformation zone, and intensifies lattice disorder, thereby promoting chip formation and material removal in a single pass.

**Figure 3:**
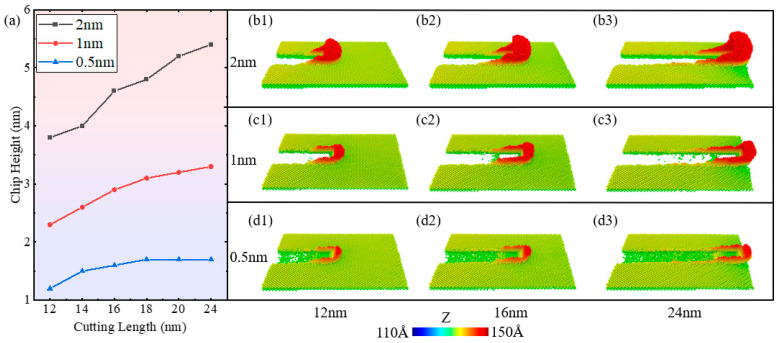
Surface morphologies of monocrystalline silicon under different cutting depths in ultra-precision machining: (**a**) statistical results of chip height; (**b1**–**b3**) cutting depth of 2 nm; (**c1**–**c3**) cutting depth of 1 nm; (**d1**–**d3**) cutting depth of 0.5 nm.

Figure 3 presents the surface morphology of single-crystal silicon after cutting at depths of 2 nm, 1 nm, and 0.5 nm. As shown in Figure 3(b1–d3), the maximum chip accumulation height increases with cutting distance, indicating that surface pile-up develops progressively during the cutting process. Under the same cutting stroke, the chip height is positively correlated with cutting depth. In particular, the chip accumulation height at a cutting depth of 2 nm is considerably larger than those obtained at 1 nm and 0.5 nm. This trend is quantitatively confirmed in Figure 3a. Mechanistically, cutting depth directly determines the volume of material involved in a single removal event. A larger cutting depth activates more atomic layers beneath the tool, expands the deformation zone, and enhances the lateral extrusion of silicon atoms. Consequently, more atoms are displaced toward the groove sides, resulting in higher and more irregular chip accumulation. By contrast, a smaller cutting depth limits the range of atomic disturbance and promotes a more localized removal process, thereby reducing chip height and improving surface uniformity. Furthermore, recent comprehensive reviews and critical trials on related advanced hard–brittle matrices, such as ceramic matrix composites and silicon carbide structures, systematically confirm that adopting a repeated shallow multi-pass removal strategy effectively transitions the chip formation mechanics toward a stable plastic flow regime. This operational configuration successfully constrains the peak transient loading beneath the cutter, thereby preventing microcrack propagation, suppressing brittle edge splintering or severe burring, and significantly reducing both surface roughness and final SSD depth [34].

**Figure 4:**
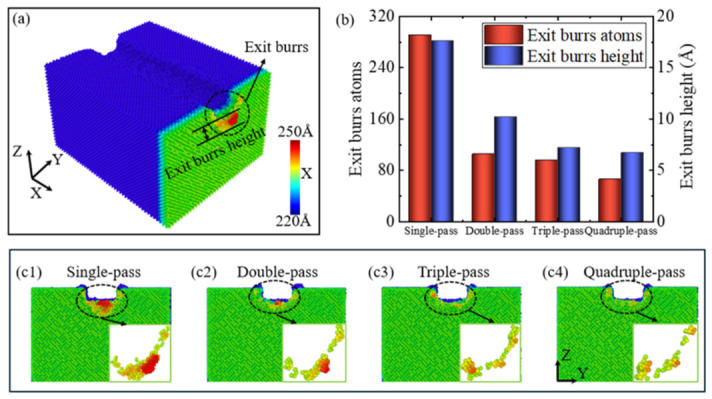
Morphologies of exit burrs of monocrystalline silicon under different cutting modes in ultra-precision machining with a fixed total cutting depth of 2 nm: d1 single-pass cutting, d2 uniform multi-pass cutting, and d3 and d4 continuous-depth gradient progressive thinning strategies: (**a**) substrate model; (**b**) statistical results of burr quantity and height; (**c1**–**c4**) surface morphologies and burrs at the exit under four different machining modes.

Figure 4 illustrates the influence of cutting mode on exit burr formation during ultra-precision machining of single-crystal silicon. The positions and heights of exit burrs are defined in Figure 4a. The surface morphologies in Figure 4(c1–c4) show that the single-pass mode d1 produces the most pronounced exit burrs, whereas the multi-pass modes suppress burr formation to varying degrees. Compared with d1, mode d2 significantly reduces the number of exit burrs, and further reductions are observed under d3 and d4. Although the visual difference between d3 and d4 is not immediately obvious from the morphology images alone, the statistical results in Figure 4b indicate that d4 further reduces both burr quantity and burr height. A similar suppression of edge pile-up was reported by Li et al. [35], who showed that regulating atomic displacement paths can promote more orderly deformation and improve the flatness of the machined surface. Decreasing the cutting depth per pass weakens the instantaneous mechanical loading at the exit boundary, thereby suppressing excessive plastic flow and improving edge quality.

**Figure 5:**
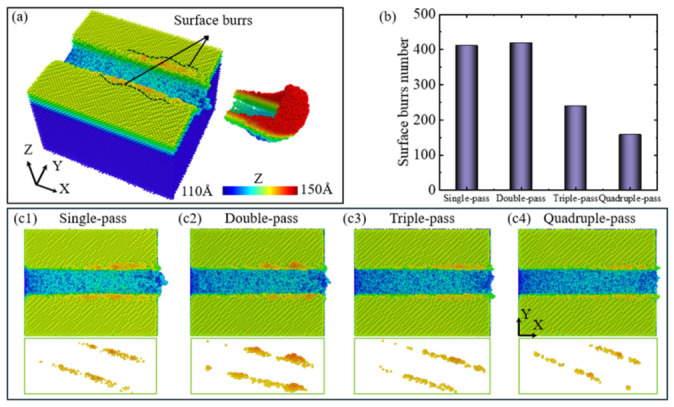
Morphologies of surface burrs of monocrystalline silicon under different machining modes in ultra-precision machining with a fixed total cutting depth of 2 nm: d1 single-pass cutting, d2 uniform multi-pass cutting, and d3 and d4 continuous-depth gradient progressive thinning strategies: (**a**) substrate model; (**b**) statistical results of surface burr quantity; (**c1**–**c4**) surface morphologies and burrs of the substrate under four different machining modes.

Figure 5 shows the evolution of surface burr morphology under different cutting modes. The locations of surface chips are indicated in Figure 5a. As shown in Figure 5(c1–c4), the morphology, distribution, and quantity of surface burrs vary markedly with the number of cutting passes. Under d1, burrs are distributed relatively uniformly on both sides of the machined groove. Under d2, the surface burr distribution becomes less uniform, with more pronounced height fluctuations and a larger burr quantity. However, when the cutting process is further divided into d3 and d4, the burr distribution becomes more regular, and the number of surface burrs decreases significantly. The statistical results in Figure 5b show that the number of surface burrs first increases and then decreases, reaching a maximum under d2. This nonmonotonic behavior suggests that simply increasing the number of passes does not necessarily lead to immediate improvement in all surface characteristics. During the first pass, burrs are mainly generated by lateral plastic flow of the undeformed surface layer. In the subsequent pass, the previously machined surface contains residual deformation, atomic disorder, and local height fluctuations, which may intensify unstable atom displacement and burr proliferation. When the number of passes increases further, the single-pass depth becomes smaller, and each pass removes only a thinner surface layer. This reduces the severity of local deformation and stabilizes atomic flow near the groove edges. Therefore, d3 and d4 provide better control of surface burr formation, with d4 showing the most favorable surface consistency.

**Figure 6:**
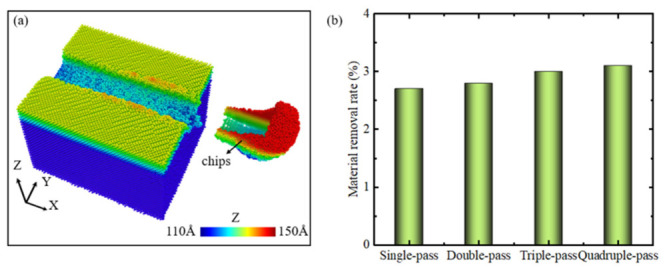
Morphologies of chips formed by monocrystalline silicon under different machining modes in ultra-precision machining with a fixed total cutting depth of 2 nm: d1 single-pass cutting, d2 uniform multi-pass cutting, and d3 and d4 continuous-depth gradient progressive thinning strategies: (**a**) substrate model; (**b**) statistical results of atomic removal rate.

Figure 6 presents the statistical results of material removal under different cutting modes. Figure 6a shows the workpiece model after machining, where removed atoms and residual chips can be identified. As shown in Figure 6b, the measured material removal rate increases with the number of cutting passes under the same total nominal cutting depth of 2 nm. This result indicates that multi-pass cutting can improve the stability and completeness of material removal. In the single-pass mode, the large cutting depth produces a high instantaneous load, which promotes severe lattice distortion and localized accumulation of deformed atoms. Part of the material may be extruded and piled up near the groove rather than being removed effectively. In contrast, the multi-pass strategy divides the total removal depth into several thinner layers. Each pass removes a smaller amount of material, which helps release residual stress, reduce excessive atomic pile-up, and convert unstable fracture-dominated removal into a more controllable shear-dominated removal process. Therefore, the improved removal rate under multi-pass cutting can be attributed to more stable layer-by-layer removal and reduced interference from severely deformed residual material.

**Figure 7:**
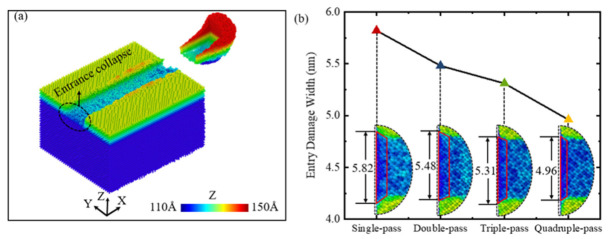
Morphologies at the entrance of monocrystalline silicon under different machining modes in ultra-precision machining with a fixed total cutting depth of 2 nm: d1 single-pass cutting, d2 uniform multi-pass cutting, and d3 and d4 continuous-depth gradient progressive thinning strategies: (**a**) substrate model; (**b**) statistical results of entrance damage width. The red border indicates the boundary used to measure the entrance damage width, and the triangles with different colors denote different cutting strategies: red for d1, blue for d2, green for d3, and yellow for d4.

Figure 7 displays the entrance morphology of single-crystal silicon under different cutting modes. Figure 7a shows the machined workpiece near the entrance region, and Figure 7b quantitatively compares the entrance damage width. Under the same total cutting depth, the entrance damage width decreases gradually as the number of cutting passes increases, from 5.82 nm under d1 to 5.48 nm under d2, 5.31 nm under d3, and 4.96 nm under d4. This trend indicates that multi-pass cutting can effectively suppress entrance damage propagation and improve the machining quality of the entrance region. In the single-pass mode, the tool immediately penetrates to the full depth of 2 nm, generating a high local contact stress and severe lattice disturbance at the entrance. This promotes lateral cracking, atom extrusion, and edge damage. By contrast, in d2, d3, and d4, the reduced single-pass depth lowers the instantaneous force imposed on the surface layer and restricts the affected region during each cutting pass. As a result, the entrance damage width is progressively reduced. These findings confirm that dividing the total cutting depth into multiple shallow passes is beneficial for improving surface integrity, especially in suppressing edge damage and stabilizing the morphology of machined silicon.

### 3.2. Mechanical Response Analysis

**Figure 8:**
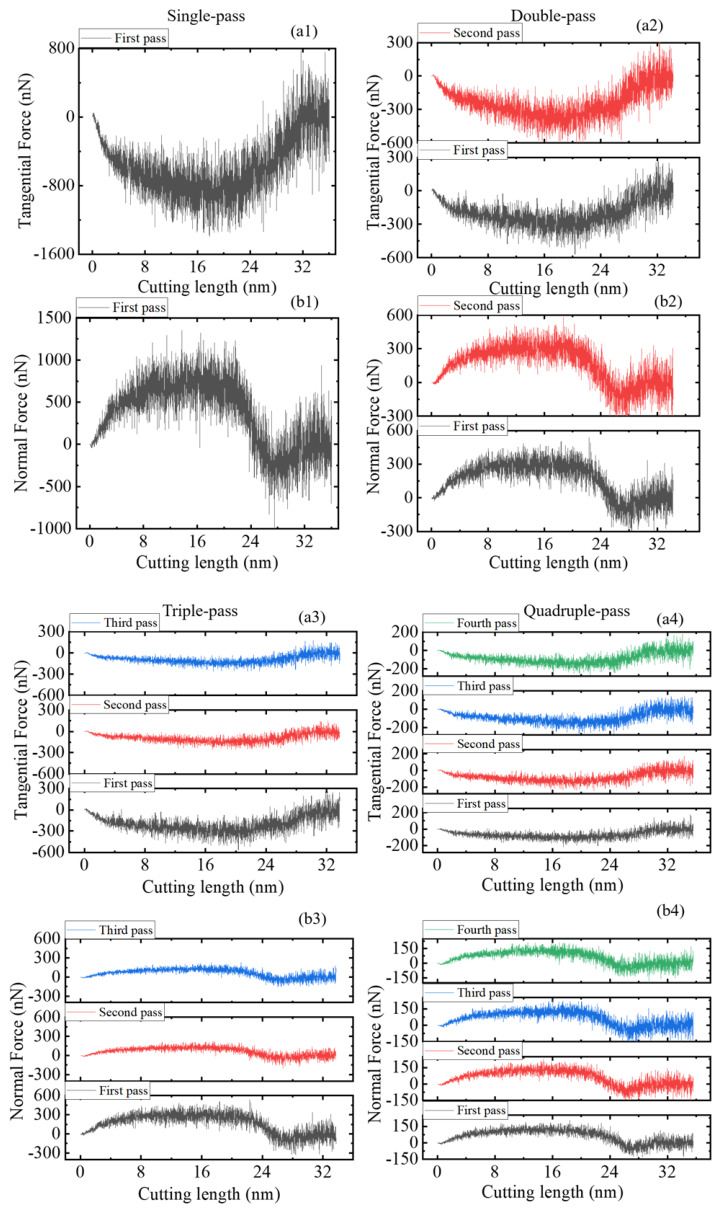
Force–distance curves of the monocrystalline silicon substrate under different cutting modes and passes with a fixed total cutting depth of 2 nm: d1 single-pass cutting, d2 uniform multi-pass cutting, and d3 and d4 continuous-depth gradient progressive thinning strategies: (**a1**–**a4**) tangential force *Fx*; (**b1**–**b4**) normal force *Fz*.

Figure 8 shows the evolution of the tangential force (*Fx*) and normal force (*Fz*) of the silicon workpiece as a function of cutting distance under different cutting modes. As shown in Figure 8(a1–a4), the tangential force exhibits a typical staged variation during cutting. In the initial cutting stage, the diamond tool first contacts the workpiece surface, and *Fx* increases rapidly because of strong surface extrusion, adhesion, and frictional resistance. As the tool further penetrates into the silicon substrate, the dominant interaction gradually changes from surface friction to plastic deformation and material removal. After this transition, the tangential force enters a relatively stable fluctuation stage. When the cutting distance exceeds approximately 20 nm, *Fx* increases slowly in some cutting modes. This increase can be attributed to the continuous accumulation of chips ahead of the tool and the progressive development of residual deformation in the machined region, both of which increase the resistance to tool advancement.

The normal force (*Fz*) in Figure 8(b1–b4) also shows a staged response. During the initial tool indentation stage, *Fz* rises rapidly as a result of strong repulsive interaction between the cutting edge and the surface atoms. With further tool movement, the normal force gradually stabilizes or decreases, indicating that the contact state between the tool and workpiece becomes more stable after the initial penetration stage. Compared with the single-pass mode d1, the force curves of the multi-pass modes show smaller fluctuation amplitudes for both *Fx* and *Fz*. This indicates that reducing the cutting depth per pass can effectively weaken instantaneous mechanical loading and suppress unstable force oscillations during material removal. Under the same total nominal cutting depth, multi-pass cutting distributes the material removal process over several shallower passes, thereby reducing the severity of local atomic extrusion and improving cutting stability. This trend is consistent with previous multistep nanogrinding studies [36], in which smaller depths of cut were found to reduce irregular mechanical fluctuations during material removal.

**Figure 9:**
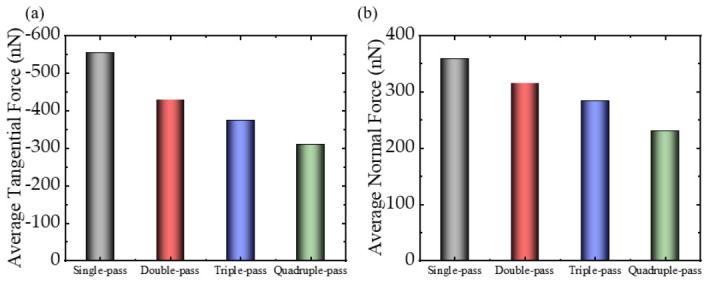
Statistical results of weighted-average forces of the monocrystalline silicon substrate varying with distance under different cutting modes with a fixed total cutting depth of 2 nm: d1 single-pass cutting, d2 uniform multi-pass cutting, and d3 and d4 continuous-depth gradient progressive thinning strategies: (**a**) weighted-average tangential force; (**b**) weighted-average normal force. The gray, red, blue, and green bars correspond to d1, d2, d3, and d4, respectively.

Figure 9 presents the weighted-average values of *Fx* and *Fz* under different cutting modes. As shown in Figure 9a,b, both the weighted-average tangential force and normal force decrease in the order of d1 > d2 > d3 > d4 under the same total cutting depth. This result indicates that increasing the number of cutting passes reduces the instantaneous mechanical load applied in each pass, partly because a smaller amount of material is engaged during each individual cutting event. Therefore, the average-force comparison primarily characterizes the force level and cutting stability rather than the mechanical efficiency per unit of removed material. The reduction in cutting force is closely related to the improvement in surface morphology discussed above. Therefore, the multi-pass strategy improves machining stability not only by decreasing the mean cutting force but also by suppressing force fluctuations during tool advancement.

Overall, decomposing the total removal depth effectively reduces cutting forces and suppresses severe local deformation, providing a stable mechanical basis for mitigating subsequent damage.

### 3.3. Subsurface Defect Structure and Stress Analysis

**Figure 10:**
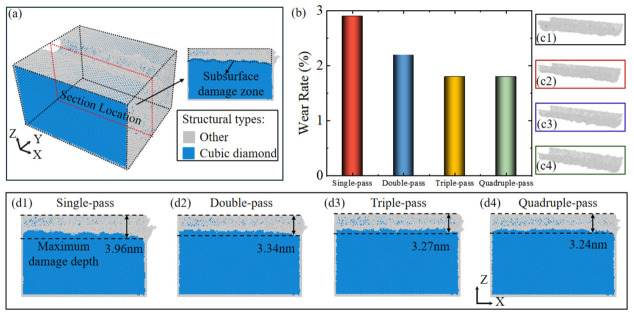
SSD of monocrystalline silicon under different cutting modes in ultra-precision machining with a fixed total cutting depth of 2 nm: d1 single-pass cutting, d2 uniform multi-pass cutting, and d3 and d4 continuous-depth gradient progressive thinning strategies: (**a**) cross-sectional schematic of the substrate model; (**b**) statistical results of damage rate; (**c1**–**c4**) SSD models; (**d1**–**d4**) cross-sectional views in the X–Z direction. The red dashed line indicates the section location used for the X–Z cross-sectional SSD analysis. The colors of the bar graphs and the borders in (**c1**–**c4**) correspond to the different cutting strategies: red for d1, blue for d2, yellow for d3, and green for d4.

Figure 10 shows the SSD characteristics of single-crystal silicon under different cutting modes during ultra-precision machining [37]. Figure 10a presents the workpiece model and marks the region used for SSD evaluation. Specifically, damaged atoms are defined as those whose local lattice configuration deviates from the perfect cubic diamond structure, which are identified as “Other” structural types. Meanwhile, the evaluation region is located directly beneath the post-machined groove. The X–Z cross-sectional morphologies in Figure 10(d1–d4) show that, under the same total nominal cutting depth, mode d1 produces a much deeper damaged layer than the three multi-pass modes, and the maximum damage is located at the exit edge of the cut, whereas the differences in damage depth among d2, d3, and d4 are relatively small. This indicates that reducing the cutting depth per pass can effectively limit the downward propagation of lattice disorder and suppress the formation of a deep SSD layer.

The three dimensional damaged regions shown in Figure 10(c1–c4) further confirm this trend. As quantified in Figure 10b, the SSD rate was calculated from the ratio of the number of damaged atoms to the original number of atoms in the selected evaluation region. Atoms retaining the cubic-diamond structure were regarded as undamaged atoms, whereas atoms classified as “Other,” whose local environments no longer corresponded to the original cubic-diamond lattice, were defined as damaged atoms. Under the same total cutting depth, the SSD rate decreases as the number of cutting passes increases, and the values for d3 and d4 are nearly identical. This result is consistent with the force response discussed in Figure 9a,b, where smaller and more stable cutting forces were obtained under multi-pass cutting. A lower mechanical load in each pass reduces the severity of lattice distortion, amorphization, and plastic deformation introduced into the subsurface region. In addition, subsequent shallow passes can partially remove or redistribute the previously deformed surface layer, leading to a more uniform stress state and a more stable material flow near the machined surface. Therefore, multi-pass cutting is beneficial for reducing the final SSD rate and improving subsurface integrity.

**Figure 11:**
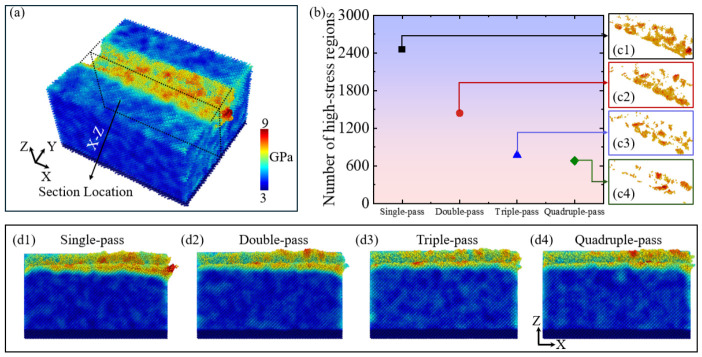
Local von Mises stress distributions of monocrystalline silicon under different cutting modes in ultra-precision machining with a fixed total cutting depth of 2 nm: d1 single-pass cutting, d2 uniform multi-pass cutting, and d3 and d4 continuous-depth gradient progressive thinning strategies: (**a**) substrate model; (**b**) statistical results of atom quantity with stress ≥ 7 GPa, the square, circle, triangle, and diamond denote d1, d2, d3, and d4, respectively; (**c1**–**c4**) models of regions with stress ≥ 7 GPa; (**d1**–**d4**) cross-sectional views in the X–Z direction.

Figure 11 presents the local von Mises stress distribution in single-crystal silicon under different cutting modes. Figure 11a shows the workpiece model, with the stress range of 3–9 GPa used as a reference for stress visualization. The X–Z cross-sectional stress maps in Figure 11(d1–d4) reveal that d1 produces a much deeper high-stress region than the multi-pass modes under the same total cutting depth. The three-dimensional distributions of atoms with von Mises stress equal to or higher than 7 GPa are shown in Figure 11(c1–c4), and the corresponding statistical results are given in Figure 11b. The threshold of 7 GPa lies within the upper-stress range of the atomic von Mises stress distribution and was selected as a consistent operational criterion for identifying regions of pronounced local stress concentration. As the number of cutting passes increases, the number of high-stress atoms decreases progressively, indicating that multi-pass cutting effectively alleviates local stress concentration. A combined comparison of Figure 4, Figure 9 and Figure 11 shows that the weighted-average tangential and normal forces, the number of atoms with von Mises stress equal to or higher than 7 GPa, and the exit burr quantity and height exhibit the same overall decreasing order from d1 to d4. This consistent trend indicates that reduced instantaneous mechanical loading and weaker local stress concentration are associated with the suppression of material pile-up and tearing at the exit boundary.

This stress redistribution is closely related to the surface morphology and burr formation behavior discussed above. Exit burrs are mainly induced by severe plastic flow and local material instability near the free boundary. When the stress concentration is strong, a larger amount of deformation energy is available to drive atomic extrusion, pile-up, and localized fracture at the exit edge. By reducing the instantaneous cutting load and lowering the number of high-stress atoms, multi-pass cutting weakens excessive plastic flow at the boundary, which explains the reduction in burr quantity and height observed in Figure 4(c1–c4). Stress reduction in multi-pass cutting stems from lower per-pass stress concentrations and progressive stress redistribution, which jointly improve surface and subsurface integrity.

**Figure 12:**
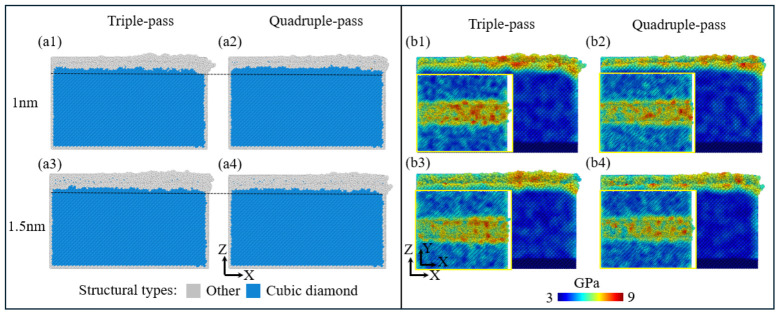
Comparison of monocrystalline silicon under d3 triple-pass and d4 quadruple-pass continuous-depth gradient progressive thinning cutting modes at the same depths of 1 nm and 1.5 nm in ultra-precision machining: (**a1**–**a4**) SSD; (**b1**–**b4**) local von Mises stress distributions.

Figure 12 further compares the SSD and stress distribution characteristics of d3 and d4 at cutting depths of 1 nm and 1.5 nm. Dai and Peng [13] reported that relieving local structural stress fields can suppress the formation of high-pressure phase atoms and related lattice defects, thereby reducing the depth of the SSD layer. As shown in Figure 12(a1–a4), at a cutting depth of 1 nm, the SSD region of d4 is smaller than that of d3. When the cutting depth increases to 1.5 nm, however, the difference between d3 and d4 becomes less pronounced, and the damaged regions tend to be similar in size. In contrast, the high-stress distributions in Figure 12(b1–b4) show that the high-stress region of d4 remains smaller than that of d3 at both cutting depths. This suggests that, even when the final SSD depth becomes comparable, reducing the cutting depth per pass can still alleviate local stress concentration and improve the stability of the machined surface.

Overall, the multi-pass strategy provides a more stable deformation environment, controlling both surface burr formation and SSD evolution through progressive stress redistribution.

## 4. Conclusions

In this study, the ultra-precision machining process of single-crystal silicon was investigated using MD simulations to elucidate the effects of cutting strategies on material removal behavior and SSD evolution. The results show that the cutting strategy significantly affects the machined surface quality and mechanical response. Increasing the number of cutting passes leads to a noticeable reduction in the average cutting force and promotes a more stable material removal process under the same total nominal cutting depth. SSD is mainly characterized by lattice distortion, amorphous transformation, and local structural disorder. Although the SSD depth shows limited variation among the multi-pass cutting modes, single-pass deep cutting produces more severe SSD, whereas increasing the cutting depth directly enlarges the damaged region. The interaction between high-stress atom clusters and the free surface plays a key role in determining the final surface integrity. The internal von Mises stress distribution further confirms that the plastic deformation zone extends ahead of the cutting tool, where high stress concentrations exceeding 7 GPa promote lattice disordering and defect formation. Under single-pass deep cutting, the more intense stress concentration induces complex defect accumulation and subsurface instability. Based on the combined analysis of surface morphology, cutting force, SSD, and stress distribution, a multi-pass cutting strategy with a single-pass depth not exceeding 1 nm is suggested to achieve a favorable balance between machining efficiency and surface integrity. Additionally, although the absolute values of cutting forces and stress may be influenced by strain-rate effects at 100 m/s, the comparative advantages of the multi-pass strategy remain consistent and independent of the specific cutting speed. These findings provide atomistic insights and theoretical guidance for the ultra-precision machining of single-crystal silicon components.

## Data Availability

Data will be made available on request.
